# The effect of performance-based financing on maternal healthcare use in Burundi: a two-wave pooled cross-sectional analysis

**DOI:** 10.1080/16549716.2017.1327241

**Published:** 2017-06-15

**Authors:** Martin Rudasingwa, Robert Soeters, Olivier Basenya

**Affiliations:** ^a^ Institute of Health Economics and Clinical Epidemiology, University Hospital of Cologne, Faculty of Medicine, University of Cologne, Cologne, Germany; ^b^ Sina Health, Den Haag, The Netherlands; ^c^ PBF Unit, Ministry of Health, Bujumbura, Burundi

**Keywords:** Performance-based financing, incentives, maternal care, Burundi, Africa

## Abstract

**Background**: Several developing countries, especially in Africa, have implemented performance-based financing (PBF) schemes with the aim of improving healthcare provision. PBF was first implemented in Burundi in 2006 as a pilot programme in three provinces and was rolled out nationwide in 2010.

**Objective**: To enrich existing studies on Burundi in three ways. Firstly, by evaluating the effect of PBF on maternal care at primary and hospital levels; secondly, on the possession of maternity logbooks for maternal care records; and thirdly, how the amount of subsidies influences healthcare outputs.

**Design**: We used data from repeated cross-sectional surveys in 500 households (intervention group: 225; control group: 275) conducted in 2006 and 2008. A total of 274 women, aged 15–49, who had recently given birth, were interviewed about the use of maternal healthcare and the possession of maternity logbooks. We performed a difference-in-differences analysis using pooled cross-sectional survey data from 2006 and 2008.

**Results**: We found that PBF is associated with an increased institutional deliveries probability of 39.5 percentage points (*p* < 0.01) – a relative improvement of 81.8%. Institutional deliveries probability increased significantly only at health centre level by 33.6 percentage points (*p* < 0.01), a  relative rise of 80.6%. There is an indication of a positive spillover effect of PBF on the possession of maternity logbooks. We found no PBF effect on the number of antenatal care visits and anti-tetanus immunization.

**Conclusions**: Our findings suggest that institutional delivery highly improved because it came from a low baseline and its unit payment was relatively high, leading health workers to promote its use. The fact that deliveries mainly increased in health centres and not in hospitals may be explained by the context of how health delivery is organized in Burundi. Health policymakers have to determine the appropriate financial incentives that best influence the improvement of each health service.

## Background

The performance of healthcare provision in many African countries is poor. The main reasons may be a scarcity of resources such as finance, infrastructure and a qualified workforce [1–3]. Yet performance is also hampered by problems in policymaking, healthcare organization and the poor motivation of health personnel [4,5]. All this results in a poor quality of care and a high mortality rate, often from treatable health conditions. The Sub-Saharan region, where Burundi is located, has a very high maternal mortality rate [6,7] of 500 deaths per 100,000 live births, compared to Asia, Latin America and the Caribbean, and developed countries which register 150, 80 and 16 deaths per 100,000 live births, respectively [8]. In an attempt to redress the aforementioned shortcomings in healthcare provision, performance-based financing (PBF) has been implemented since the early 2000s in many African countries.

PBF for healthcare provision, when well-tailored to the context of implementation, has shown to be a promising reform approach to improving the utilization and the quality of care [9–11]. The aim of PBF is the improvement of healthcare provision by motivating healthcare providers and steering them to increase their efforts and technical efficiency. In a narrower sense, PBF aims at improving the use and quality of care, as well as providing additional financial resources to health facilities necessary for the improvement of that care’s delivery [12,13]. Since around 2000, more than 30 African countries have implemented, or are currently piloting, PBF schemes [9]. Under such schemes, health providers receive financial incentives for results in terms of health services rendered and their quality of care. PBF incentives have the potential to motivate health personnel to improve healthcare delivery. Yet, other non-monetary incentives in the PBF approach are also important such as improving transparent management through the introduction of business plans and financial management tools. Other characteristics of the PBF approach are to enhance the autonomy of health facilities and to promote good governance through the separation of roles according to the main functions in health systems. These functions are: regulation, provision, contract development and verification, payment and strengthening consumer influence [13].

Deliveries attended by qualified staff in the health facility, as well as antenatal and postnatal care are seen as crucial in guarding against maternal mortality [14]. Thus, PBF schemes link incentives to the aforementioned maternity services with the aim of fostering their use and quality. Several original and review studies have shown the implementation of PBF helped to increase the number of deliveries in health facilities and the improvement of the quality of antenatal care (ANC) [15–21]. A literature review by Morgan et al. [15] suggests that financial incentives have great potential in improving the quantity and quality of maternal health services. Some studies in Rwanda only found an increase in institutional deliveries, but no increase in ANC use by pregnant women [22,23]. A study by Gertler and Vermeersch [12] in Rwanda found that performance incentives helped health providers to improve their adherence to appropriate clinical practices by approximately 20%. Despite the promising effect of performance-based incentives on improving care quality, evidence of the PBF effect in other studies was mixed, with both improvements and non-improvements found in incentive-supported health services [16,24,25]. The success of PBF interventions also depends on the context in which a programme operates [9]. Notably, it is important to apply the full set of PBF best practices such as competition for contracts, involvement of the private sector, separation of functions and the promotion of autonomous management in health facilities [13]. Notwithstanding the mixed evidence of PBF, this strategy is seen as novel, with the potential to spur health providers to achieve predefined health targets linked to financial incentives [15]. More research into the effectiveness of PBF on different aspects of healthcare delivery is warranted.

This study analysed the effect of PBF on maternal health services in Burundi. It used data from performance evaluations of healthcare provision in 2006 at PBF baseline and from 2008 after two years of follow-up. The data were gathered in the two provinces of Bubanza and Cankuzo with a PBF scheme (intervention group) and in the two provinces of Karuzi and Makamba without a PBF scheme (control group). A study of PBF in Burundi by Bonfrer and colleagues [21] included three maternal health services of institutional deliveries, at least one antenatal visit and at least one tetanus vaccination. Bonfrer and colleagues showed that PBF was only associated with positive effects on institutional deliveries (an increase of 36%). However, they did not show to which health facilities (health centre or hospital) and to which qualified staff (physician or nurse) the PBF effect was linked. In Burundi, maternal services in health facilities are performed in health centres or in hospitals, and are provided by either physicians or nurses. The quality of care in health centres is judged to be lower than in hospitals and the clinical knowledge of nurses is lower than that of physicians. Analysing in which health facilities maternal health services were performed and which medical staff provided them would indicate the ‘real value’ of the PBF effect on these services. The documentation of maternal health services is crucial for follow-up and further healthcare (e.g. prenatal care and detection of possible complications). However, to the best of our knowledge, the PBF effect on the possession of maternity logbooks in Burundi has not yet been analysed. In Burundi, the PBF incentive for a caesarean section was 10 times higher than for a normal (vaginal) birth. One may suspect that the higher incentive would induce a higher number of caesarean sections. Hence, the importance of assessing this. Another important variable that the present paper aims to analyse is the effect of PBF on full tetanus immunization. In Burundi, as in other many African countries, tetanus immunization coverage for pregnant women is still at a low level, thus putting pregnant women at risk of death [26]. To increase the coverage of tetanus immunization among pregnant women, health providers receive a financial incentive if all recommended tetanus vaccines have been administered. Yet, the effect of PBF on full tetanus immunization for pregnant women in Burundi is still unknown. The present paper seeks to close the aforementioned gaps in our knowledge of the PBF effect on maternal health services in Burundi.

## Methods

### The PBF scheme in Burundi

In 2006, the Burundian Government, in collaboration with international non-governmental organizations (NGOs), piloted PBF schemes in three provinces: Bubanza, Cankuzo and Gitega. Two other provinces served as control settings: Karuzi and Makamba [27]. The Burundian Health Ministry chose the two groups of PBF and non-PBF health facilities based on the socio-economic similarities of the affiliated provinces. The non-PBF health facilities continued to use the traditional input-based financing. The PBF programme aimed at improving the weak utilization and poor quality of health services, including maternal health services [28]. In 2010, Burundi rolled out the PBF scheme at national level, the second African country to do so after Rwanda [19,21]. The health facilities received financial incentives based on the quantity and quality of the health services provided.

Incentive payments were based on the volume of health services rendered (quantity) and qualitative performance scores (quality). The quality indicators were developed during the preparation of the PBF programme in collaboration with the Ministry of Health. The health facilities reported the quantity of the incentivized health services monthly in their routine Health Management Information System (HMIS); the figures were then independently verified. The quality of care was assessed quarterly by an evaluation team from the district and provincial health authorities. This produced a score, which was taken into consideration for the payment of a quality bonus. The quality of the incentivized health services was assessed using a health indicator quality-checklist. The quantity and quality of health services were verified and validated between 2006 and 2010 by international NGOs that supported the Burundian Government in implementing the PBF scheme. From 2010 onwards, this role was taken over by a semi-autonomous verification team at provincial level with members from the Burundian health authorities, local territorial administration and civil society, advised by international NGO staff. As of 2010, the PBF incentives accounted, on average, for 20% of the total health facility revenues [27]. Moreover, in 2013 this proportion increased to around 40% [29].

For the volume of health services provided, each quantitative health indicator had a fixed amount of money or subsidy attached to it and the total payment was calculated by multiplying the number of cases by the unit payment of that indicator. The financial incentive from the quality indicators (quality bonus) was paid quarterly and calculated as follows:

(1)
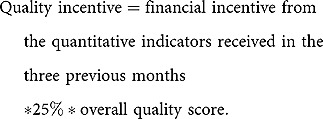

In 2010, the formula to calculate the quality bonus changed with the aim of penalizing poorly performing health facilities. Based on the obtained quality scores, the bonus reward has changed as follows [27]: (1) quality score higher than 70%, a maximum quality bonus of 25% of the financial bonus from the quantitative indicators is rewarded; (2) quality score between 50% and 70%, there is no financial bonus; (3) quality score between 50% and 60%, corrective actions must be taken to improve the quality of care; (4) quality score below 50%, urgent corrective actions must be taken to improve the quality of care and administrative sanctions may be taken (e.g. the director of the health facility may be dismissed). Furthermore, the health facility is penalized by losing 25% of the financial bonus from the quantitative performance.

Table 1 gives an example of unit payments for some maternal health services. The PBF scheme in Burundi, and also in other African countries, due to lack of sufficient financial resources, does not aim to provide incentives that cover the full costs of healthcare provision. The main aim of PBF is to motivate healthcare providers to make more efforts. The other sources of income such as government subsides, out-of-pocket and insurance payments, and partner contributions as well as potential moral hazard on the part of healthcare providers are also taken into account in calculating PBF bonuses [13]. For instance, in Burundi, the costs of a vaginal birth were estimated in 2006 at around USD 10 and for a caesarean at more than USD 100, whereas their unit PBF bonuses were USD 2 and USD 20, respectively. Such small incentives that do not enable providers to cover the costs of healthcare production, especially in the case of the free health services, might be a demotivational factor for healthcare providers to make greater efforts as desired.

**Table 1.** Unit payments or subsidies for the maternal health services.

Maternal output indicators	Bonus per unit in US$
Pregnant woman fully immunized	0.50
Antenatal care: new and standard visits	0.40
Institutional delivery by qualified staff (normal birth)	2.00
Institutional delivery by qualified staff (caesarean)	20.00

Source: based on [27,p.77]

### Sample and data collection

Randomly selected households were surveyed in the two provinces of Bubanza and Cankuzo with PBF (intervention group) and in the two provinces of Karuzi and Makamba without PBF (control group). The intervention and control provinces were selected based on their similarities in terms of socio-economic indicators and health characteristics. Repeated cross-sectional household surveys were conducted in 2006 and 2008 in both intervention and control provinces, and included 500 households (intervention group: n = 225; control group: n = 275). The four provinces encompassed 77 catchment areas (a catchment area is defined as the area supply of one health centre). Among these 77 catchment areas, 20 catchment areas were randomly selected and afterwards 25 households were randomly selected in each of the 20 catchment areas, which resulted in a total of 500 households which were surveyed in both 2006 and 2008 (see Table 2 for detail on the randomization of households).

**Table 2.** Randomization of households.

Study groups	Provinces	Population	Catchment areas	Randomly selected catchment areas	Randomly selected households
Intervention group	Bubanza	313,000	19	5	125
	Cankuzo	190,000	20	4	100
	Subtotal	503,000	39	9	225
Control group	Karusi	365,000	20	6	150
	Makamba	311,000	18	5	125
	Subtotal	676,000	38	11	275
	Total	1,179,000	77	20	500

The study design was a prospective experimental study. The household surveys collected data on the socio-demographic characteristics of the population and use of different health services. This paper focuses on the use of maternal health services in women aged between 15 and 49. The data collection was conducted by a team of 10 experienced investigators and 2 supervisors, in both the PBF and non-PBF households. In 2006, the survey was conducted between 17 June and 1 July while in 2008, the survey was conducted between 5 and 17 June. Before each survey – both in 2006 and in 2008 – the investigators received four days of training on how to collect the study data. The pilot study received authorization from the Burundian Ministry of Health. Informed consent was obtained from the respondents.

### Outcome measures

The incentivized outcome measures relate to ANC visits, anti-tetanus immunization and institutional delivery. The possession of an ANC card was included to indicate whether women possessed a ‘maternity logbook’ as a variable of maternity records. To qualify for PBF incentives, the ANC and the delivery should be carried out in a health facility by a qualified member of staff (physician or nurse). Health facilities only received financial bonuses if a pregnant woman was fully immunized. In health centres, nurses provide health services, while in hospitals there are also physicians. The PBF incentives per ANC visit, per anti-tetanus immunization and per normal childbirth were the same in health centres as in hospitals. However, the quality of care is judged to be better in hospitals than in health centres. As a quality indicator of care provided, we included a variable showing whether the women used ANC or gave birth in a hospital or in a health centre. The survey data provide information from women who used one to three ANC visits or who did not use any at all. For ANC, we created two dependent variables: one indicating whether the woman used at least one ANC visit and the other dependent variable indicating if a woman used three ANC visits (the maximum number of ANC visits considered during the pilot study). For the incentivized maternal immunization against tetanus, we created two variables; one indicates whether the woman had at least one tetanus vaccine and the other variable whether the mother was fully vaccinated. Institutional delivery shows whether a woman gave birth in a health facility assisted by a qualified member of staff. Additionally, we included a variable as to whether the birthing mother was assisted by a doctor or a nurse in a health centre or in a hospital. Caesarean sections were incentivized with a 10 times higher subsidy compared to normal births. We included a variable indicating whether the delivery was a normal birth or caesarean.

## Analysis

We first analysed the performance change between 2006 and 2008 in each outcome measure and this was assessed separately in health facilities with and without financial incentives. Later we performed a difference-in-differences (DD) estimation to assess the effect of the PBF financial incentives by calculating the difference between the changes in mean outcomes in the intervention and control groups between the pre- and post-implementation times:

(2)
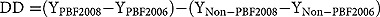

A DD model was run for each outcome of interest as follows:

(3)
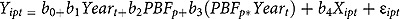

where i denotes the individual, 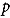
the province and *t* is the year (2006 as baseline and 2008 as evaluation year). 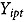, the dependent variables, are the outcome measures for individual i, in province 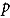
and in year *t*. 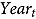
is a dummy variable for baseline and post-implementation time (2008 = 1 and 2006 = 0). 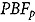
is a dummy variable indicating whether the facility where the mother received maternal services was in the PBF group or not (PBF = 1 for intervention provinces and 0 for control provinces). 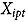
is a vector of covariates. The variable of interest is 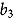, the interaction term between PBF and implementation year, which shows the net effect of PBF financial incentives (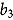
indicates the value of the PBF effect). The term 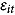
is the random error assumed to be normally distributed. To account for multiple testing issues and the small sample, Bonferroni correction [30] for the descriptive analysis was used. Due to there being few clusters, the heteroscedasticity-consistent standard errors for the inference robustness of the DD model [31,32] were used instead of using cluster-robust standard errors. IBM SPSS Statistics version 22 was used and the statistical significance level was set at 5%.

## Results

Comparison of the intervention and control provinces at baseline indicates the similarity of the two groups in terms of socio-economic and demographic characteristics (Table 3 and Table A1). The absolute changes of maternal health services between 2006 and 2008 (Table 4) indicate that in the control group, there was no significant improvement in any of the maternal health services. However, in the intervention group, significant improvements were observed in institutional delivery with those in possession of maternity logbooks for ANC demonstrating percentage-point changes of 39.2 and 17.7, respectively. The institutional deliveries improved in the intervention provinces, in both health centres and hospitals, with 29.1 and 10 percentage-point changes, respectively. Only the deliveries assisted by nurses improved significantly in the intervention provinces, by 27.7 percentage points. The majority of the ANC and deliveries, in both the intervention and control groups, occurred in health centres. Nurses assisted the majority of the deliveries in all the studied provinces. For ANC visits and anti-tetanus vaccinations, there were no significant changes (at least one ANC visit, three or more ANC visits, ANC in hospitals or health centres, at least one tetanus vaccination, and full tetanus immunization) in both the intervention and control groups. There were no significant changes in the delivery methods (normal or caesarean) in either the intervention or control group. Although the subsidy per caesarean delivery was 10 times higher than per a normal delivery, there was no significant increase in caesarean delivery in the intervention group.

**Table 3.** Baseline controls characteristics.

Characteristics	Intervention	Control	Diff.	*p*-value (two-tailed)
Household size (mean [SD])	6.2 (2.4)	6.8 (2.2)	–0.6	0.141
No education (%)	45.0%	31.4%	0.136	0.113
Health insurance (%)	3.3%	8.6%	–0.053	0.281
Married (%)	96.7%	92.9%	0.038	0.341
Number of children (mean [SD])	1.6 (1.5)	1.9 (1.4)	0.3	0.230
Farmer (%)	85.0%	81.4%	0.036	0.592
Ownership of fertile land (%)	67.3%	78.8%	–0.115	0.153
Very poor (%)	18.3%	22.9%	–0.046	0.526
Poor (%)	20.0%	32.9%	–0.129	0.099
Less poor (%)	25.0%	25.7%	–0.007	0.926
Adequate home lighting (%)	78.3%	67.1%	0.106	0.155

**Table 4.** Percentage of women reporting key maternal services.

Intervention group (n = 132)			Control group (n = 142)			
Key maternal services	2006^1^	2008	Absolute change^2^	2006^1^	2008	Absolute change^2^
At least one ANC visit	96.7	100.0	3.3	98.6	98.6	0.0
Three or more ANC visits	80.0	83.3	3.3	80.0	70.8	–9.2
ANC in health facility						
Hospital^3^	3.3	8.3	5.0	1.4	1.4	0.0
Health centre	93.3	91.7	–1.6	97.1	97.2	0.1
ANC maternity logbook	76.7	94.4	17.7**	87.1	88.9	1.8
At least one tetanus vaccination	95.0	98.6	3.6	91.4	95.8	4.4
Full tetanus immunization	70.0	68.1	–1.9	70.0	62.5	–7.5
Institutional delivery	48.3	87.5	39.2***	72.9	73.6	0.7
Delivery in health facility						
Hospital^3^	6.7	16.7	10.0	12.9	13.4	0.5
Health centre	41.7	70.8	29.1*	60.0	59.7	–0.3
Delivery assisted by						
Physician^3^	3.3	8.3	5.0	2.9	0.0	–2.9
Nurse	41.7	69.4	27.7**	64.3	69.4	5.1
Delivery method						
Normal childbirth	96.7	94.4	–2.3	100	100	0.0
Caesarean section^3^	3.3	5.6	2.3	0.0	0.0	0.0

*Note*s: ANC: antenatal care. ^1^Except for institutional delivery (*p* = 0.004), no statistical differences found between intervention and control groups at baseline. ^2^T-test for differences between 2006 and 2008. *p = 0.002, **p = 0.001, ***p < 0.001 (two-tailed *p*-values based on Bonferroni correction and bootstrapping method). ^3^ The numbers of ANC visits and deliveries in hospitals, the numbers of deliveries assisted by physicians, and of Caesarean sections were insufficient to calculate the test statistics.

For all the pregnant women in the intervention group, 93.3% and 91.7% used ANC in health centres at least once in 2006 and 2008, respectively. In the control group, for all the expectant mothers, around 97% used ANC at least once in health centres in 2006 and 2008. Only 3.3% and 8.3% of pregnant women used ANC in hospital in 2006 and 2008, respectively. Likewise, most of the institutional deliveries occurred in health centres: 41.7% and 70.8% of the expectant mothers in the intervention group in 2006 and 2008, respectively, against 6.7% and 16.7% in hospitals in 2006 and 2008, respectively. In the control group, there were no big changes between 2006 and 2008 with nearly 60% of deliveries occurring in health centres in 2006 and 2008 and nearly 13% in 2006 and 2008 in hospitals. The nurses assisted the majority of the deliveries assisted by qualified staff. In the intervention group, nurses assisted 41.7% and 69.4% of the deliveries in 2006 and 2008, respectively. Physicians assisted 3.3% and 8.3% of the deliveries in 2006 and 2008, respectively. In the control group, the nurses assisted 64.3% and 69.4% of the deliveries in 2006 and 2008, respectively. Physicians assisted 2.9% in 2006. In 2008 no delivery was assisted by a physician in the control group.

The results of the DD estimator indicate the effect of PBF (Table 5 and Table A2 for full DD estimates). The DD coefficients show the difference in maternal care use between health facilities with PBF and health facilities without PBF that might be attributable to PBF. The findings show that the probability of a woman giving birth in a PBF health facility improved by 39.5 percentage points (*p* = 0.001), an increase of 81.8%. The institutional delivery in health centres was statistically significantly different between the intervention and the control group. The probability of delivery in PBF health centres increased by 33.6 percentage points (*p* = 0.008), an increase of 80.6%. The possession of maternity logbooks and the delivery assisted by nurses improved, however without a PBF effect at a significance level of 5%. The probability of possession of maternity logbooks improved by 16.5 percentage points (*p* = 0.068), an increase of 21.5%. The probability of delivery assisted by nurses improved by 22.6 percentage points (*p* = 0.053), an increase of 54.2%. No PBF effect on ANC, tetanus immunization and delivery method was found. Healthcare ownership by the women as an endogenous variable was not included in the covariate variables; however, its non-inclusion does not change the statistically significant variables, but the coefficients of the dependent variables slightly increase. There is no difference in terms of statistically significant variables when estimating the DD model with or without the control variables. Including the control variables increases the statistical inference of the DD estimates.

**Table 5.** Estimated effect of PBF on maternal health care use.

Maternal care use (n = 274)	ß	(SE)*	*p*-value*	% improvement**
Any ANC visit	0.044	(0.031)	0.1575	4.5%
Three or more ANC visits	0.128	(0.104)	0.223	16%
Maternity logbook	0.165	(0.090)	0.068	21.5%
At least one tetanus vaccination	0.008	(0.061)	0.895	0.8%
Full tetanus immunization	0.100	(0.104)	0.816	14.3%
ANC in health centres	–0.036	(0.044)	0.415	–3.8%
Institutional delivery	0.395	(0.111)	0.001	81.8%
Delivery in health centres	0.336	(0.125)	0.008	80.6%
Delivery assisted by nurses	0.226	(0.117)	0.053	54.2%
Normal delivery	–0.023	(0.036)	0.519	–2.4%

*Note*s: ANC: antenatal care; SE: standard errors. *Standard errors and two-tailed *p*-values calculated using heteroscedasticity-consistent SE method. **% improvement in intervention group = (ß/baseline mean) x 100. All models include the control variables listed in Table 3 and provinces and year (time) controls.

## Discussion

The findings of our study suggest that PBF led to an increase in institutional delivery in the relatively short time period of two years. This increase in institutional delivery was significant for the deliveries in health centres compared to those in hospitals and was also significant for the deliveries assisted by nurses compared to physicians. However, the findings suggest that PBF had no effect on ANC use or anti-tetanus vaccination. From the descriptive analysis (Table 4), the possession of maternity logbooks and the deliveries assisted by nurses highly increased in PBF health facilities (*p* = 0.001) from 76.7% in 2006 to 94.4% in 2008, and from 41.7% in 2006 to 69.4% in 2008, respectively. However, our findings could not explicitly attribute this increase to PBF. The increase of the possession of maternity logbooks may have resulted from a Hawthorne effect which might be viewed as a positive spillover effect of PBF. The increase of deliveries assisted by nurses in the intervention group suggests that the number of deliveries per nurse increased, which may lead to work overload if the number of nurses is not sufficient. The work overload may negatively affect the quality of care and the nurses’ motivation. A study on PBF in Burundi by Falisse et al. [33] found a larger increase of qualified nurses in PBF provinces (197%) than in non-PBF provinces (124%) between 2005 and 2009. However, since the findings of the current study show that nearly all deliveries are assisted by nurses, further robust research is needed to assess the relation between the use of institutional delivery care and the number of qualified nurses.

The significant increase of institutional delivery and not of ANC and tetanus vaccination could be explained by the fact that ANC already started from a high baseline. Additionally, the relatively higher financial incentives of $2 per normal childbirth compared to the financial incentives of ANC ($0.40) and tetanus vaccination ($0.50) may be another explanation of the increase of institutional deliveries. The health providers might have made more effort to sensitize pregnant women towards giving birth in health facilities during the prenatal visits. Health facilities also partnered with community health workers as agents who passed through the communities encouraging women to find their nearest health facilities in good time before giving birth [34]. The non-increase in ANC use may also be caused by the fact that use of antenatal and anti-tetanus services during pregnancy are more related to cultural and behavioural aspects (e.g. low knowledge about the importance of antenatal consultations and vaccines and beliefs that vaccines are not safe and not needed) and could be better addressed by interventions focused at community level [35]. To qualify for PBF subsidies for an anti-tetanus vaccination, a pregnant woman had to be fully immunized – meaning all the required anti-tetanus injections had to be administered. The difficulty in achieving this may be a reason why PBF had little effect on full immunization against tetanus. For the variables of having at least one ANC visit and at least one tetanus vaccination, their mean rate values at baseline were very high (96.7% and 95%, respectively) – indeed so high there was no room for a significant increase. Contrary to our findings, a study by Falisse et al. [33] used routine data from the Burundian National Health Information System (NHIS) and found an effect of PBF on anti-tetanus vaccinations of 20 percentage points. However, the study does not specify if it was a full or a partial anti-tetanus vaccination.

The significant increase of institutional deliveries in health centres is explained by the way healthcare is organized in Burundi. In Burundi health centres play the role of ‘gatekeepers’ that provide a premium package of healthcare, only sending complicated cases to hospitals for additional, high complementary care. Patients can also freely go to hospitals for outpatient treatments without referral from health centres, but the costs of treatment in hospitals are higher than those of treatments in health centres. Thus, most patients go to health centres. Hospitals compared to health centres are few in number and located far from the villages where the majority of people live. This also explains why more health services are provided in health centres than in hospitals. During the pilot period of the PBF scheme in Burundi, the government introduced free care for deliveries and healthcare for children under five years old nationwide, including the intervention and the control provinces [36]. Therefore, the increase in institutional deliveries in the intervention group is explained by the financial incentives provided to health providers. The healthcare providers possibly made more effort to sensitize and encourage expectant mothers to give birth in health facilities. One may suggest the increase in institutional deliveries was due to the interaction between the free delivery in facilities and the PBF. However, in Rwanda, where institutional deliveries are not free, PBF has also stimulated an increase in deliveries at the facilities [19]. In Burundi, nurses constitute the largest proportion of qualified staff. The Burundian Ministry of Health report 2013 indicates in Burundi there was one physician per 18,335 people and one nurse per 1395 people [37]. Thus, in a PBF scheme or not, most institutional deliveries will be assisted by nurses.

The subsidy for a caesarean delivery was 10 times higher than the subsidy for a normal delivery, so one could expect more unnecessary caesarean deliveries as a result of ‘gaming’. Yet, this was not the case. This is probably because hospitals could not make a large profit with a subsidy of $20 for a caesarean section. This finding underlines the importance of monitoring the effect of subsidies on the results and avoiding perverse incentives, based on the level of target achievements and other contextual factors in the implementation area. The subsidy per indicator should be high enough to create its desired effect, but not so high that, for example, physicians will prefer a caesarean section for monetary gain above a normal delivery [13].

Previous studies in the neighboring countries of Burundi such as Rwanda and the Democratic Republic of Congo have also suggested a positive effect of PBF on institutional deliveries. However, as in other studies carried out in Burundi, they do not show to which health facilities and which qualified staff the PBF effect was linked [18,19,38]. The findings of those studies also suggested the high financial bonus per childbirth was the main reason for the increase in institutional deliveries. A study carried out in Burundi indicated that the PBF had a positive effect on the quality of maternal health services in terms of structure and process quality indicators [39]. Based on the findings of the current study, that PBF had a positive effect on institutional deliveries provided in health centres where care is delivered by nurses, it would be interesting to know the quality of the health outcomes, such as maternal and newborn mortality and life-threatening complications during, or shortly after, childbirth. This should be analysed in further research as it could not be assessed using our data-set and based on our best knowledge, there are no previous study data available.

The study has some limitations. First, based on the data-set at our disposal, it was not possible to incorporate all the important determinants of healthcare-seeking behaviour in our analysis. These would have helped to determine if the difference in the increase of childbirth and getting full ANC and anti-tetanus immunization was caused only by the differences in unit payments or caused by some differences in healthcare-seeking behaviours. This should be assessed in further research. Second, from a total of 17 provinces of Burundi, PBF was only piloted in 3 provinces and another 2 provinces served as the control group. The data-set of the current study was only collected in two provinces with PBF implementation. The findings of the current study may not represent the situation in all provinces. However, since there is no significant difference in terms of healthcare provision between the different provinces in Burundi, the findings of this study may still generate lessons learned for the implementation of PBF in other provinces and even in health settings of other countries.

Notwithstanding these limitations, the findings of this study show that PBF may constitute an appropriate strategy to reverse the situation in many developing countries where healthcare use, especially in childbirth and other maternal health services, remains at a low level.

## Conclusion

The findings of our study indicate that the PBF approach in Burundi has led to the increase of the institutional deliveries carried out in health centres under the assistance of nurses. Thus, to improve the quality of maternal health services, the main focus should lie in strengthening health centres in healthcare provision in terms of incentives and capacity building. Although the PBF bonus unit of a cesarean section was 10 times higher than for a normal childbirth, almost all institutional deliveries were normal births. There is an indication of a positive spillover effect of PBF on the possession of maternity logbooks in the intervention group. There was no effect on ANC visits and anti-tetanus injections, which already at the baseline were at a higher level, and with relatively lower unit bonuses. PBF designers and policymakers will have to determine a financial bonus that is appropriate to motivate healthcare providers to make more effort towards improving healthcare provision.

## Paper context

Performance-based financing (PBF) in Burundi has helped to increase institutional delivery rates. Only deliveries assisted by nurses in health centres increased. No PBF effect was found on incentivized antenatal care use and anti-tetanus immunization and on non-incentivized possession of maternity logbooks. The reasons for these mixed results on incentivized services are suggested to be the differences in unit incentive payments and the baseline performance scores of these health services. An appropriate and context-based financial incentive system is recommended.

## Appendix

**Table A1.** Baseline characteristics of health centres.

	Intervention (n = 9)	Control (n = 10)	Diff.	*p*-value
Number of beds (mean [SD])	11 (2.449)	13.40 (11.491)	–2.4	0.548
Public facility (mean [SD])	0.88 (0.333)	0.7 (0.483)	0.18	0.341
Number of nurses A1 and A2 (mean [SD])	1.11 (0.601)	1.30 (1.494)	–0.19	0.728
Number of nurses A3 (mean [SD])	2.33 (1.414)	3.0 (2.0)	–0.67	0.418
Number of non-qualified staff (mean [SD])	7.89 (2.934)	10.70 (3.302)	–2.81	0.068
Log total budget (BUFR)	13.58 (0.311)	13.80 (0.556)	–0.22	0.326
Log total population	9.66 (0.345)	9.81 (0.514)	–0.15	0.475

A1: a 3-year university degree; A2: a 4-year secondary school degree after 9-year primary school (new education system); A3: a 2-year secondary school degree after 4-year basic (general) secondary school (old education system).

**Table A2.** Full difference-in-differences results.

	Any ANC visit (=1)	Three or more ANC visits (=1)	Maternity logbooks (=1)	At least one tetanus vaccination (=1)	Full tetanus vaccination (=1)	ANC in health centres (=1)	Institutional delivery (=1)	Delivery in health centres (=1)	Delivery assisted by nurses (=1)	Normal delivery (=1)
Treatment*post	0.044 (0.031)	0.128 (0.104)	0.165* (0.090)	0.008 (0.061)	0.100 (0.123)	–0.036 (0.044)	0.395*** (0.111)	0.336*** (0.125)	0.226* (0.117)	–0.023 (0.036)
Treatment (=1)	–0.021 (0.033)	–0.022 (0.777)	–0.081 (0.075)	0.031 (0.050)	0.020 (0.086)	–0.010 (0.026)	–0.282*** (0.086)	–0.202** (0.90)	–0.226*** (0.087)	–0.030 (0.025)
Post (2008) (=1)	–0.006 (0.016)	–0.082 (0.074)	0.017 (0.060)	0.026 (0.045)	–0.112 (0.083)	–0.010 (0.023)	0.009 (0.075)	–0.029 (0.087)	0.052 (0.080)	0.002 (0.006)
Household size	–0.010 (0.008)	–0.012 (0.021)	–0.012 (0.017)	–0.016 (0.012)	–0.020 (0.025)	0.001 (0.008)	–0.013 (0.023)	–0.011 (0.025)		–0.002 (0.007)
No education (=1)	0.003 (0.015)	0.234*** (0.071)	–0.010 (0.061)	–0.030 (0.037)	–0.089 (0.089)	0.013 (0.037)	–0.105 (0.085)	–0.112 (0.096)		0.022 (0.028)
Married (=1)	–0.010 (0.014)	–0.028 (0.100)	–0.001 (0.070)	–0.051* (0.021)	–0.186 (0.096)	–0.040* (0.020)	0.054 (0.122)	–0.005 (0.124)		0.034 (0.051)
Number of children	0.018 (0.010)	0.093** (0.036)	0.037 (0.030)	0.013 (0.022)	0.013 (0.044)	0.009 (0.013)	–0.005 (0.042)	–0.019 (0.044)		0.006 (0.009)
Farmer (=1)	–0.015 (0.012)	–0.048 (0.066)	–0.047 (0.055)	0.002 (0.041)	0.019 (0.084)	–0.045 (0.044)	0.035 (0.077)	0.002 (0.087)		–0.015 (0.029)
Ownership of fertile land (=1)	–0.011 (0.012)	0.003 (0.054)	0.011 (0.048)	0.013 (0.029)	–0.064 (0.062)	–0.009 (0.026)	0.0145 (0.060)	–0.059 (0.066)		0.006 (0.020)
Very poor (=1)	0.011 (0.028)	–0.119 (0.092)	0.035 (0.073)	–0.002 (0.048)	0.112 (0.099)	0.035 (0.045)	–0.053 (0.088)	0.040 (0.104)		0.019 (0.029)
Poor (=1)	0.007 (0.028)	–0.041 (0.078)	0.084 (0.062)	–0.028 (0.050)	0.119 (0.089)	0.058 (0.035)	–0.174** (0.084)	–0.003 (0.095)		0.032 (0.022)
Less poor (=1)	0.003 (0.024)	–0.052 (0.074)	0.028 (0.068)	–0.025 (0.040)	0.178* (0.084)	0.020 (0.049)	–0.044 (0.077)	0.032 (0.091)		0.005 (0.035)
Adequate home lighting (=1)	0.021 (0.021)	0.032 (0.066)	–0.022 (0.048)	0.023 (0.034)	0.041 (0.073)	0.011 (0.027)	0.143** (0.067)	0.149* (0.074)		–0.010 (0.017)

*Note*: ****p* < 0.01, ***p* < 0.05, **p* < 0.1.
